# Betulinic Acid Suppresses UBE2T Expression via MAPK/ERK Inhibition to Block FANCI and FANCD2 Monoubiquitination in Glioblastoma

**DOI:** 10.1111/jcmm.71000

**Published:** 2026-01-04

**Authors:** Yifeng Bao, Maode Wang

**Affiliations:** ^1^ Department of Neurosurgery The First Affiliated Hospital of Xi'an Jiaotong University Xi'an China; ^2^ Department of Neurosurgery Huashan Hospital, Fudan University Shanghai China; ^3^ Center of Brain Science The First Affiliated Hospital of Xi'an Jiaotong University Xi'an China

**Keywords:** betulinic acid, DNA damage repair, Fanconi anaemia pathway, glioblastoma, MAPK/ERK, UBE2T

## Abstract

Platinum‐based chemotherapy remains a cornerstone of glioma treatment, yet resistance driven by the Fanconi anaemia (FA) DNA repair pathway limits efficacy. Here, we identified betulinic acid (BA) as a potent inhibitor of FA pathway activation. BA pretreatment abrogated cisplatin‐induced monoubiquitination of FANCI/FANCD2 and disrupted their nuclear foci formation and interactions with downstream repair proteins (ERCC1, REV1 and BRCA1), leading to persistent DNA interstrand crosslinks without affecting intrastrand lesion repair. Biochemical analyses revealed that BA selectively suppressed UBE2T expression at the transcriptional level, without altering mRNA stability or protein degradation, thereby blocking the FANCL‐UBE2T‐mediated ID2 monoubiquitination cascade. In vivo, BA significantly enhanced the antitumour efficacy of cisplatin in xenograft models. Mechanistically, BA inhibited MAPK/ERK signalling, and pharmacological reactivation of ERK reversed BA‐induced suppression of UBE2T and tumour growth. Collectively, these findings uncover a previously unrecognised MAPK/ERK‐UBE2T‐FA axis in glioma and highlight BA as a potential adjuvant to overcome cisplatin resistance through transcriptional repression of UBE2T.

## 1 Introduction

Glioblastoma (GBM) is one of the most aggressive primary brain tumours and is well recognised for its poor prognosis, frequent recurrence, and profound therapeutic resistance [1]. Despite advances in surgical resection, radiotherapy, chemotherapy, and emerging immunotherapies, survival outcomes for patients with high‐grade GBM remain dismal [2]. Cisplatin and other platinum‐based agents remain important components of treatment; however, many tumours display intrinsic resistance or acquire tolerance during therapy, greatly limiting clinical benefit [3, 4]. Understanding the molecular drivers of this resistance is therefore critical for developing more effective treatment strategies.

Among DNA repair systems, the Fanconi anaemia (FA) pathway plays a pivotal role in maintaining genome stability by resolving DNA interstrand crosslinks generated by platinum compounds [5, 6]. Activation of the FA pathway requires the E2 ubiquitin‐conjugating enzyme UBE2T, which cooperates with the E3 ligase FANCL to catalyse monoubiquitination of FANCI and FANCD2, thereby initiating repair through homologous recombination (HR) [7, 8]. In glioblastoma, elevated FA pathway activity, particularly through UBE2T‐dependent regulation, has been associated with enhanced repair capacity and reduced responsiveness to DNA‐damaging therapies, positioning this pathway as a potential target to overcome chemoresistance [9, 10, 11].

Betulinic acid (BA), a natural pentacyclic triterpenoid isolated from birch bark, has been extensively investigated for its anticancer activities [12, 13]. In glioblastoma, BA has been shown to inhibit tumour growth by inducing apoptosis, promoting reactive oxygen species (ROS) accumulation, and suppressing oncogenic signalling cascades [14, 15, 16]. Beyond its intrinsic cytotoxic effects, BA has also been reported to enhance the efficacy of conventional chemotherapeutic agents [17, 18]. In particular, several studies indicate that BA can sensitise tumour cells to cisplatin by aggravating DNA damage, amplifying oxidative stress, and impairing pro‐survival signalling [19, 20]. These findings suggest that BA functions not only as a direct antitumour agent but also as a potential chemosensitiser, with particular relevance to glioblastoma biology and platinum‐based chemotherapy. However, whether BA directly modulates the DNA repair machinery remains largely unexplored.

In this study, we uncovered a previously unrecognised mechanism by which betulinic acid enhances cisplatin sensitivity in glioblastoma. Our data demonstrate that betulinic acid suppresses the transcriptional expression of UBE2T, the E2 ubiquitin‐conjugating enzyme essential for the Fanconi anaemia (FA) core complex, thereby preventing monoubiquitination of the FANCD2‐FANCI complex and weakening FA pathway activity. As the FA pathway plays a central role in the repair of interstrand crosslinks and the development of chemoresistance, these findings broaden the current understanding of betulinic acid's molecular actions and highlight UBE2T suppression as a novel strategy to impair DNA repair and improve therapeutic response in glioblastoma.

## 2 Materials and Methods

### 2.1 Cell Culture

The human glioblastoma cell lines U87MG (ATCC Cat. No. HTB‐14), U251 (MilliporeSigma, Cat. No. 09063001), and LN229 (ATCC Cat. No. CRL‐2611) were used in this study. U87MG and LN229 cells were cultured in Dulbecco's Modified Eagle Medium (DMEM; Gibco, Thermo Fisher Scientific) supplemented with 10% (v/v) fetal bovine serum (FBS; Gibco) and 1% penicillin–streptomycin (Gibco). U251 cells were maintained in Minimum Essential Medium (MEM; Gibco) with 10% FBS and 1% penicillin–streptomycin. All cell lines were grown at 37°C in a humidified incubator containing 5% CO_2_. Cell line authenticity was verified by short tandem repeat (STR) profiling within the last 3 years, and routine mycoplasma testing confirmed the absence of contamination before experimental use.

For drug treatment, cisplatin (MedChemExpress, Cat. No. HY‐17394) was used at 10 μM for 12 h. BA (MedChemExpress, Cat. No. HY‐10529) was applied as a 24 h pretreatment at 20 μM prior to subsequent assays. The USP1 inhibitor ML323 (MedChemExpress, Cat. No. HY‐12782) was used at 5 μM for 4 h. Actinomycin D (MedChemExpress, Cat. No. HY‐17559) was applied at 5 μg/mL for the indicated times to block transcription. Cycloheximide (CHX; MedChemExpress, Cat. No. HY‐12320) was used at 50 μg/mL for protein stability assays. MG132 (MedChemExpress, Cat. No. HY‐13259) was used at 10 μM for 6 h to inhibit proteasomal degradation. Recombinant human FasL (MedChemExpress, Cat. No. HY‐P700520) was used at 100 ng/mL for 6 h [21]. DPN (MedChemExpress, Cat. No. HY‐12452) was used at 10 nM for 4 h [22]. Recombinant human IGF‐1 (MedChemExpress, Cat. No. HY‐P70698) was used at 50 ng/mL for 30 min [23]. ERK agonist Phorbol Myristate Acetate (PMA; MedChemExpress, Cat. No. HY‐18739) was used at 100 nM for 1 h [24]. Recombinant human DLL4‐Fc (Abcam, Cat. No. ab108557) was used at 2 μg/mL overnight [25]. Cells were harvested at the indicated times after treatment for protein or RNA analyses.

### 2.2 Western Blot

Cell lysates were obtained using RIPA buffer. Nuclear proteins were extracted using the NE‐PER Nuclear and Cytoplasmic Extraction Reagents kit (Thermo Fisher Scientific, Cat. No. 78833) according to the manufacturer's protocol, and nuclear fractions were collected for Western blot analysis. Protein concentrations were determined with the BCA Protein Assay Kit (Thermo Fisher Scientific, Cat. No. 23227). Equal amounts of protein (40 μg per sample) were resolved by SDS‐PAGE and transferred onto PVDF membranes. Membranes were then blocked with 5% non‐fat milk prepared in TBST, followed by overnight incubation at 4°C with primary antibodies diluted 1:2000 including FANCI (Abcam, Cat. No. ab245219), FANCD2 (CST, Cat. No. 16323), ERCC1 (CST, Cat. No. 12345), REV1 (Abcam, Cat. No. ab75328), BRCA1 (CST, Cat. No. 9010), FANCA (CST, Cat. No. 14657), FANCC (Abcam, Cat. No. ab5065), FANCF (NOVUS, Cat. No. NBP1‐51916), FANCM (CST, Cat. No. 38199), FAAP24 (NOVUS, Cat. No. NBP1‐91876), FANCL (Abcam, Cat. No. ab272618), FANCB (CST, Cat. No. 14243), FAAP100 (Abcam, Cat. No. ab180157), UBE2T (CST, Cat. No. 12992), USP1 (CST, Cat. No. 8033), UAF1 (Abcam, Cat. No. ab122473), Pro‐Caspase 8 (Asp374), p43/p41 and p18 (CST, Cat. No. 9496), YY1 (CST, Cat. No. 46395), Lamin B1 (CST, Cat. No. 13435), ERβ (CST, Cat. No. 89954), pERK1/2 (Thr202/Tyr204) (CST, Cat. No. 4370), ERK1/2 (CST, Cat. No. 4695), p‐IGF‐1Rβ (Tyr1135/Tyr1136) (CST, Cat. No. 3024), IGF‐1Rβ (CST, Cat. No. 3027), Cleaved Notch 1 (CST, Cat. No. 4147), Notch 1 (CST, Cat. No. 3608), β‐Actin (CST, Cat. No. 4970). After thorough washing, the membranes were incubated for 1 h at room temperature with HRP‐conjugated secondary antibodies (anti‐rabbit IgG, CST, Cat. No. 7074; or anti‐mouse IgG, CST Cat. No. 7076), diluted 1:5000. Protein bands were subsequently detected using Clarity Western ECL Substrate (BioRad, Cat. No. 1,705,060) and quantified by densitometric analysis. β‐Actin and Lamin B1 were served as the loading control for total protein and nuclear extracts.

### 2.3 FANCD2 Foci Immunofluorescence Assay

Glioma cells were cultured on sterile glass coverslips and treated with cisplatin as indicated. To extract soluble proteins, samples were briefly incubated for 2 min in ice‐cold cytoskeleton buffer (10 mM PIPES, pH 6.8; 100 mM NaCl; 300 mM sucrose; 3 mM MgCl_2_; 1 mM EGTA; 0.5% Triton X‐100; 1 mM DTT; supplemented with 1× protease and phosphatase inhibitors). Immediately afterward, cells were fixed for 10 min at room temperature with 4% paraformaldehyde prepared in PBS. Following three rinses with PBS, permeabilization was performed with 0.5% Triton X‐100 in PBS for 5 min, and nonspecific binding was blocked for 1 h using 5% BSA in PBS. Coverslips were then incubated overnight at 4°C with an anti‐FANCD2 primary antibody (diluted 1:1000 in blocking buffer). The next day, after PBS washes, Alexa Fluor–conjugated goat anti‐mouse IgG secondary antibody (Invitrogen, Cat. No. A‐11001; 1:1000) was applied for 1 h at room temperature. Nuclear DNA was stained with DAPI (Sigma‐Aldrich, Cat. No. D9542), and samples were mounted using ProLong Gold Antifade reagent (Thermo Fisher Scientific, Cat. No. P36930). Images were acquired with a laser‐scanning confocal microscope equipped with a 60×/63× oil objective, ensuring identical exposure settings across groups. Z‐stacks were collected at 0.4 μm intervals, and maximum‐intensity projections were processed in ImageJ. For quantification, at least 100–200 nuclei per condition from three or more independent replicates were analysed in a blinded manner. Nuclei containing ≥ 5 FANCD2 foci were classified as foci‐positive. Both the proportion of positive nuclei and the average number of foci per nucleus were calculated. Statistical comparisons were performed using one‐way ANOVA followed by the appropriate post hoc analysis, as detailed in the figure legends.

### 2.4 Long‐Fragment Quantitative PCR (qPCR Blocking) Assay

This assay was applied to measure the induction and repair of DNA interstrand crosslinks. Genomic DNA was harvested from glioma cells at 0, 6, and 24 h after treatment and quantified using spectrophotometry. DNA from both treated samples and untreated controls (NC) was purified and subjected to SYBR Green‐based amplification. Primers targeting the human HBB locus were used: long‐fragment (~10.1 kb), forward 5′‐GCTGAGTTCTCTGGCTGTGTTC‐3′, reverse 5′‐CCAGGAGAAGTCAGGGTAGGAA‐3′; short fragment (~122 bp), forward 5′‐TGCACGTGGATCTGTCCGAA‐3′, reverse 5′‐GCACCTGACTCTCTCCACCA‐3′. Cycle threshold (Ct) values were used to calculate lesion frequencies: ΔCt_long = Ct_long(treated) – Ct_long(NC); ΔCt_short = Ct_short(treated) – Ct_short(NC). Relative amplification (RA) for each amplicon was obtained as 2^−ΔCt^, and normalised amplification was expressed as RA_norm = RA_long/RA_short. Assuming Poisson distribution, interstrand crosslink frequency per 10 kb was computed as λ(10 kb) = −ln(RA_norm) × (10,000/L), where L is the length of the long amplicon. To confirm specificity for crosslinks, DNA was de‐crosslinked at 65°C for 2 h in 50 mM Tris–HCl (pH 8.0), restoring amplification of the long product to baseline.

### 2.5 Dot Blot

Genomic DNA was purified from glioma cells using a phenol‐chloroform protocol, treated with RNase A, and quantified by UV absorbance. For each sample, 0.5 μg DNA was adjusted to equal volume, denatured with 0.4 M NaOH and 10 mM EDTA for 10 min at room temperature, and rapidly cooled on ice. Two microliters of the denatured DNA was spotted onto positively charged nitrocellulose membranes either manually or with a dot‐blot apparatus. Membranes were neutralised with 2× SSC, air‐dried, and cross‐linked by UV exposure (150 mJ/cm^2^, 30 min). After blocking with 5% milk/TBST for 1 h at room temperature, membranes were probed overnight at 4°C with a monoclonal antibody against cisplatin‐DNA adducts (clone ICR4, Merck, Cat. MABE416, 1:1000). Secondary HRP‐conjugated antibody (1:5000) was applied for 1 h at room temperature, and chemiluminescent signals were developed. To confirm equal loading, membranes were briefly stained with 0.02% methylene blue in 0.3 M sodium acetate (pH 5.2). Positive controls (in vitro cisplatin‐modified DNA) and negative controls (untreated DNA) were included on each blot. Signal intensities were quantified using ImageJ and normalised to total DNA staining, with results expressed relative to control.

### 2.6 Immunoprecipitation

Glioma cells (1 × 10^7^) were washed in ice‐cold PBS and lysed for 30 min on ice with immunoprecipitation buffer (50 mM Tris–HCl, pH 7.5; 150 mM NaCl; 0.5% Triton X‐100; 1 mM EDTA; supplemented with protease/phosphatase inhibitors). Supernatants were clarified by centrifugation (14,000 × g, 15 min, 4°C), and protein concentrations were determined by BCA assay. One milligram of protein lysate was pre‐cleared with Protein A/G magnetic beads (Thermo Fisher, Cat. 88,803) for 30 min at 4°C. Samples were then incubated overnight at 4°C with 1 μg anti‐FANCD2 antibody under rotation, followed by bead capture for 2 h. Beads were washed five times with lysis buffer (first two washes at 150 mM NaCl, with an optional high‐salt wash at 300 mM NaCl for stringent conditions). Bound proteins were eluted by boiling in 2× Laemmli buffer (65.8 mM Tris–HCl, pH 6.8; 2.1% SDS; 26.3% glycerol; 0.01% bromophenol blue) for 5 min. Inputs (1%) and eluates were resolved by SDS‐PAGE and transferred to PVDF membranes. Western blotting was performed with antibodies against ERCC1, REV1, and BRCA1 to detect co‐immunoprecipitated partners; FANCD2 blotting confirmed pulldown efficiency. Control IPs with isotype IgG or beads alone were included. In selected assays, lysates were supplemented with 50 μg/mL ethidium bromide to assess DNA‐mediated interactions. Quantification was carried out by densitometry, and co‐IP signals were normalised to FANCD2 recovery and to input protein levels.

### 2.7 Polysome Profiling

For ribosome profiling, cells were pretreated with cycloheximide (CHX; 100 μg/mL, 10 min, 37°C) to immobilise translating ribosomes, rinsed twice with ice‐cold CHX‐containing PBS, and lysed on ice for 10 min in polysome extraction buffer (20 mM Tris–HCl, pH 7.5; 150 mM NaCl; 5 mM MgCl_2_; 1% Triton X‐100; 0.5% sodium deoxycholate; 1 mM DTT; 100 μg/mL CHX; RNase inhibitor). Lysates were clarified by centrifugation (14,000 × g, 10 min, 4°C), and equal *A*_260_ units were loaded onto prechilled 10%–50% sucrose gradients prepared in gradient buffer (20 mM Tris–HCl, pH 7.5; 150 mM NaCl; 5 mM MgCl_2_; 1 mM DTT; 100 μg/mL CHX). Gradients were centrifuged in a Beckman SW41Ti rotor at 210,000 × g for 2 h at 4°C. Fractions (1 mL each) were collected while continuously monitoring *A*_254_ to identify 80S monosome and polysome peaks. In control gradients treated with EDTA, polysomes collapsed into monosomes, confirming accurate peak assignment.

RNA from each fraction was isolated using acid phenol–chloroform extraction, reverse‐transcribed, and analysed by RT‐qPCR to quantify UBE2T transcripts. For each sample, UBE2T mRNA levels in monosome (80S) and polysome (≥ 2 ribosomes) fractions were summed and expressed as percentages of total signal across the gradient. Data from at least three independent experiments were used to plot distribution profiles (fraction index vs. normalised transcript abundance). A shift of UBE2T mRNA from polysome to monosome fractions was interpreted as diminished translational engagement, whereas an unchanged profile indicated preserved translation initiation and elongation.

### 2.8 Quantitative PCR (qPCR)

Total RNA was extracted using Super FastPure Cell RNA Isolation Kit (Vamyme, Cat. No. RC102‐01) following the manufacturer's instructions. Complementary DNA (cDNA) was synthesised using the HiScript IV All‐in One Ultra RT SuperMix Kit (Vamyme, Cat. No. R433‐01) and was conducted with SupRealQ Ultra Hunter SYBR qPCR Master Mix (Vazyme, Cat. No. Q713‐02) on a T100 Thermal Cycler (BioRad, Cat. No. 1,861,096). The mRNA level of UBE2T was normalised to β‐Actin. Primer sequence targeted human UBE2T (NM_014176, OriGene, Cat. No. HP210681) and β‐Actin (NM_001101, OriGene, Cat. No. HP204660) were used.

### 2.9 Xenograft Mouse Model and in Vivo Drug Treatment

BALB/c nude mice (4–6 weeks old, 16–20 g) were housed under specific pathogen‐free conditions with unrestricted access to food and water. Human glioma U87MG cells (5 × 10^6^ cells in 100 μL PBS mixed 1:1 with Matrigel) were subcutaneously implanted into the right flank of each mouse. Once tumours reached approximately 5 cm^3^, animals were randomly divided into three groups (*n* = 5 per group): (1) control, receiving intraperitoneal injections of vehicle; (2) cisplatin, treated with cisplatin at 5 mg/kg intraperitoneally every 5 days; and (3) BA + cisplatin, administered BA (10 mg/kg orally every 5 days [26]) in combination with cisplatin (5 mg/kg, intraperitoneally every 5 days). Tumour volume was determined using the formula *V* = (length × width^2^)/2. After 4 weeks of treatment, mice were sacrificed, and tumours were harvested, weighed, and prepared for biochemical and histological analyses. All animal procedures were approved by the Institutional Animal Care and Use Committee (IACUC) of Huashan Hospital and carried out in accordance with institutional and national ethical guidelines.

### 2.10 Immunohistochemistry (IHC)

Xenograft tumours were excised, fixed in 10% neutral‐buffered formalin for 48 h, processed routinely and embedded in paraffin. Sections (4 μm) were deparaffinised in xylene and rehydrated through a graded ethanol series to distilled water. Endogenous peroxidase was quenched with 3% H_2_O_2_ in PBS for 10 min. Antigen retrieval was achieved by heating in citrate buffer (10 mM sodium citrate, pH 6.0) at 95°C for 15 min, followed by cooling to room temperature. After blocking with 5% normal goat serum for 30 min, sections were incubated overnight at 4°C with primary antibodies diluted in antibody diluent: UBE2T (1:200) and phospho‐ERK1/2 (1:200). Slides were washed in PBST and incubated with an HRP‐conjugated anti‐rabbit detection system for 30 min at room temperature. Signal was developed with DAB substrate (Proteintech, Cat. PR30018) and nuclei were counterstained with haematoxylin, followed by dehydration, clearing, and mounting. UBE2T and phospho‐ERK1/2 staining were quantified by the H‐score method.

### 2.11 Statistical Analysis

All data were shown as the mean ± SEM unless otherwise stated. The number of independent biological replicates (*n*) is indicated in each figure legend. Statistical significance between two groups was determined using two‐tailed unpaired Student's *t*‐tests, whereas multiple group comparisons were performed by one‐way ANOVA. Analyses were carried out with SPSS version 20.0, and differences were considered statistically significant at *p* < 0.05.

## 3 Results

### 3.1 BA Suppresses Cisplatin‐Triggered Activation of the FA Signalling Cascade in Glioma Cells

**FIGURE 1:**
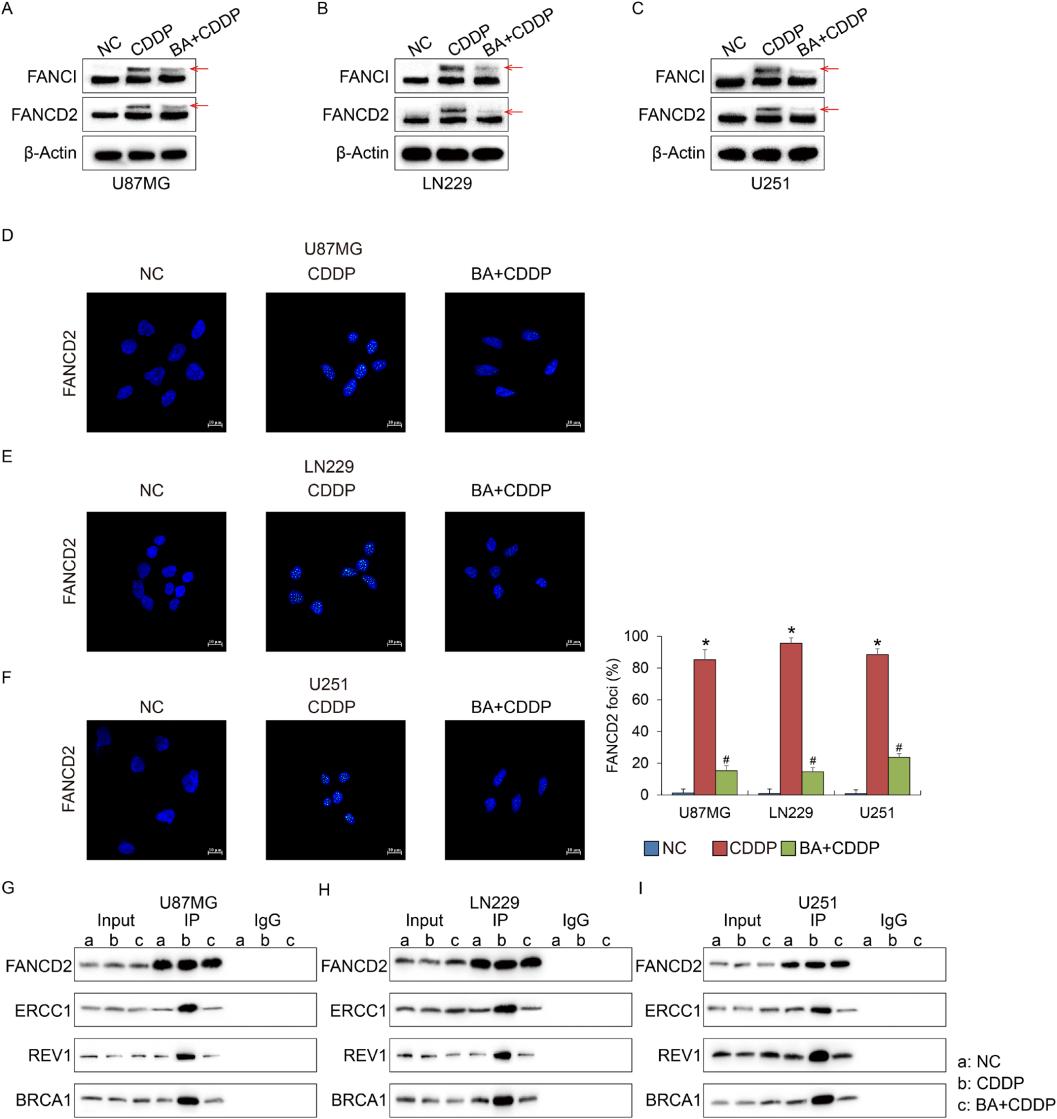
Ba suppresses FANCI/FANCD2 monoubiquitination, FANCD2 foci formation, and downstream DNA repair complex assembly in glioma cells. (A–C) Western blot analysis of FANCI and FANCD2 monoubiquitination in U87MG (A), LN229 (B), and U251 (C) cells treated with cisplatin (CDDP) or BA plus CDDP. Cisplatin induces robust monoubiquitination of both FANCI and FANCD2, whereas BA pretreatment markedly diminishes these modifications (red arrows), indicating inhibition of FA pathway activation. β‐Actin served as a loading control. (D–F) Immunofluorescence analysis show the FANCD2 nuclear foci formation in U87MG (D), LN229 (E), and U251 (F) cells. Blue: DAPI‐stained nuclei; green: FANCD2 Foci. Quantification (right) revealed a significant decrease in the percentage of FANCD2‐positive nuclei in BA + CDDP‐treated cells compared with CDDP alone. Data are presented as mean ± SEM (*n* = 3 independent experiments); *p < 0.05 vs. NC, #*p* < 0.05 vs. CDDP. (G–I) Co‐immunoprecipitation (Co‐IP) assays examining FANCD2‐associated repair proteins. In all three glioma cell lines, cisplatin enhances the interaction between FANCD2 and REV1, BRCA1 and ERCC1, reflecting activation of translesion synthesis (TLS) and homologous recombination (HR) branches of DNA repair. BA pretreatment substantially weakens these interactions, suggesting that BA not only blocks FANCD2 monoubiquitination but also disrupts the recruitment of downstream repair effectors.

To explore whether betulinic acid (BA) influences FA pathway activity, three glioma cell lines with distinct genetic and pathological features were examined: U87MG (glioblastoma multiforme, PTEN mutant, wild‐type p53, poorly differentiated), LN229 (glioblastoma, wild‐type PTEN, p53 mutant, moderately differentiated), and U251 (glioblastoma, p53 mutant, highly proliferative). When exposed to cisplatin, all three cell types exhibited robust monoubiquitination of FANCI and FANCD2 (Figure 1A–C), demonstrating that FA pathway activation is a conserved response in glioma. Strikingly, when BA was administered prior to cisplatin treatment, this modification of FANCI and FANCD2 was largely abrogated (Figure 1A–C). Consistently, immunofluorescence analysis revealed that the nuclear accumulation of FANCD2 foci was markedly reduced by BA pretreatment (Figure 1D–F). Through immunoprecipitation assays, we further assessed protein–protein interactions within the FA repair network in glioma cells. In U87MG, LN229, and U251 cells, cisplatin normally enhanced the association of FANCD2 with REV1, BRCA1 and ERCC1. However, the recruitment of them was markedly all weakened when cells were pretreated with BA (Figure 1G–I). The reduction in FANCD2 binding partners suggests that BA not only blocks FA pathway activation but also disrupts FA‐coupled downstream repair steps, including ERCC1‐dependent incision, TLS‐mediated ICL bypass and HR‐associated resolution, rather than globally impairing canonical NER, TLS, or HR pathways.

**FIGURE 2:**
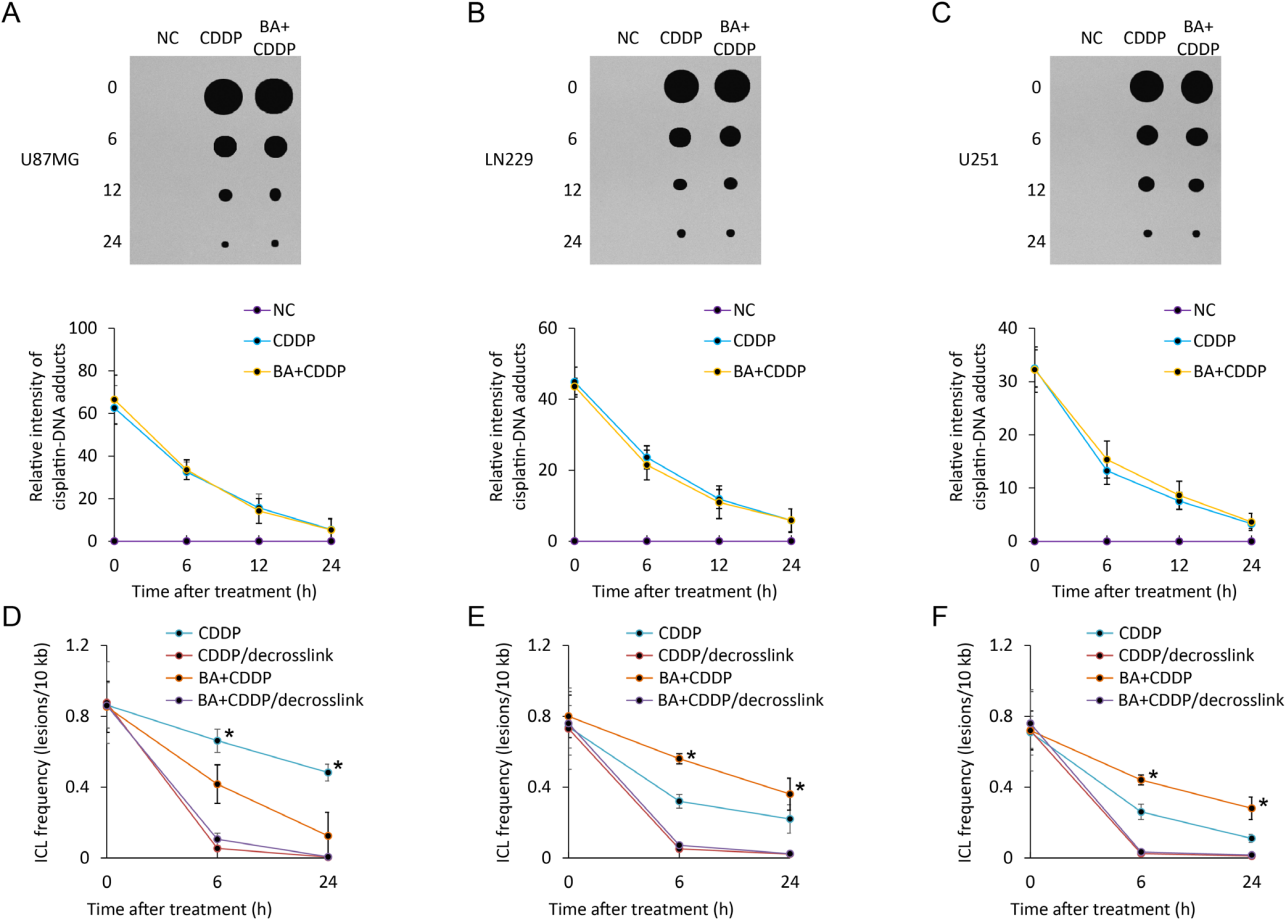
BA selectively impairs the repair of interstrand DNA crosslinks but not intrastrand adducts induced by cisplatin. (A–C) Dot blot analysis of cisplatin‐DNA intrastrand adducts in U87MG (A), LN229 (B), and U251 (C) cells treated with cisplatin (CDDP) alone or in combination with BA. The intensity of DNA adducts is quantified at the indicated recovery times (0–24 h). Comparable kinetics of intrastrand adduct removal are observed between CDDP and BA + CDDP groups, indicating that BA does not interfere with intrastrand repair. (D‐F) Long‐fragment quantitative PCR (LF‐qPCR) analysis of interstrand crosslink (ICL) repair in U87MG (D), LN229 (E), and U251 (F) cells. The lesion frequency (lesions per 10 kb) is calculated from amplification efficiency at different time points. BA pretreatment markedly delays ICL removal, reflected by the persistent lesions at 6 and 24 h post‐recovery, whereas CDDP‐treated cells exhibit efficient ICL resolution. Data are presented as mean ± SEM (*n* = 3 independent experiments); *p < 0.05 versus CDDP.

The functional consequences of this inhibition were evaluated by monitoring DNA crosslink dynamics. Dot blot assay was used to track the fate of intrastrand crosslinks, and the removal of intrastrand crosslinks occurred with comparable kinetics in BA‐treated and untreated groups (Figure 2A–C). Long‐fragment qPCR was used to examine interstrand crosslinks over time. Cisplatin alone induced both lesion types, and repair progressed as expected. Notably, cells pretreated with BA displayed a pronounced defect in the resolution of interstrand crosslinks, as shown by their prolonged persistence during the recovery period in all three glioma cell lines (Figure 2D–F). These observations indicate that BA selectively compromises the FA‐dependent repair of interstrand crosslinks while leaving intrastrand lesion repair largely unaffected. This selectivity is consistent with the observation that BA affects ERCC1 recruitment in the context of FA‐mediated ICL unhooking, but does not impair the canonical NER machinery responsible for repairing cisplatin intrastrand adducts. Collectively, our results establish BA as an inhibitor of FA pathway activation in glioma cells in vitro.

### 3.2 BA Suppresses UBE2T Transcription Through MAPK/ERK Pathway Inhibition

**FIGURE 3:**
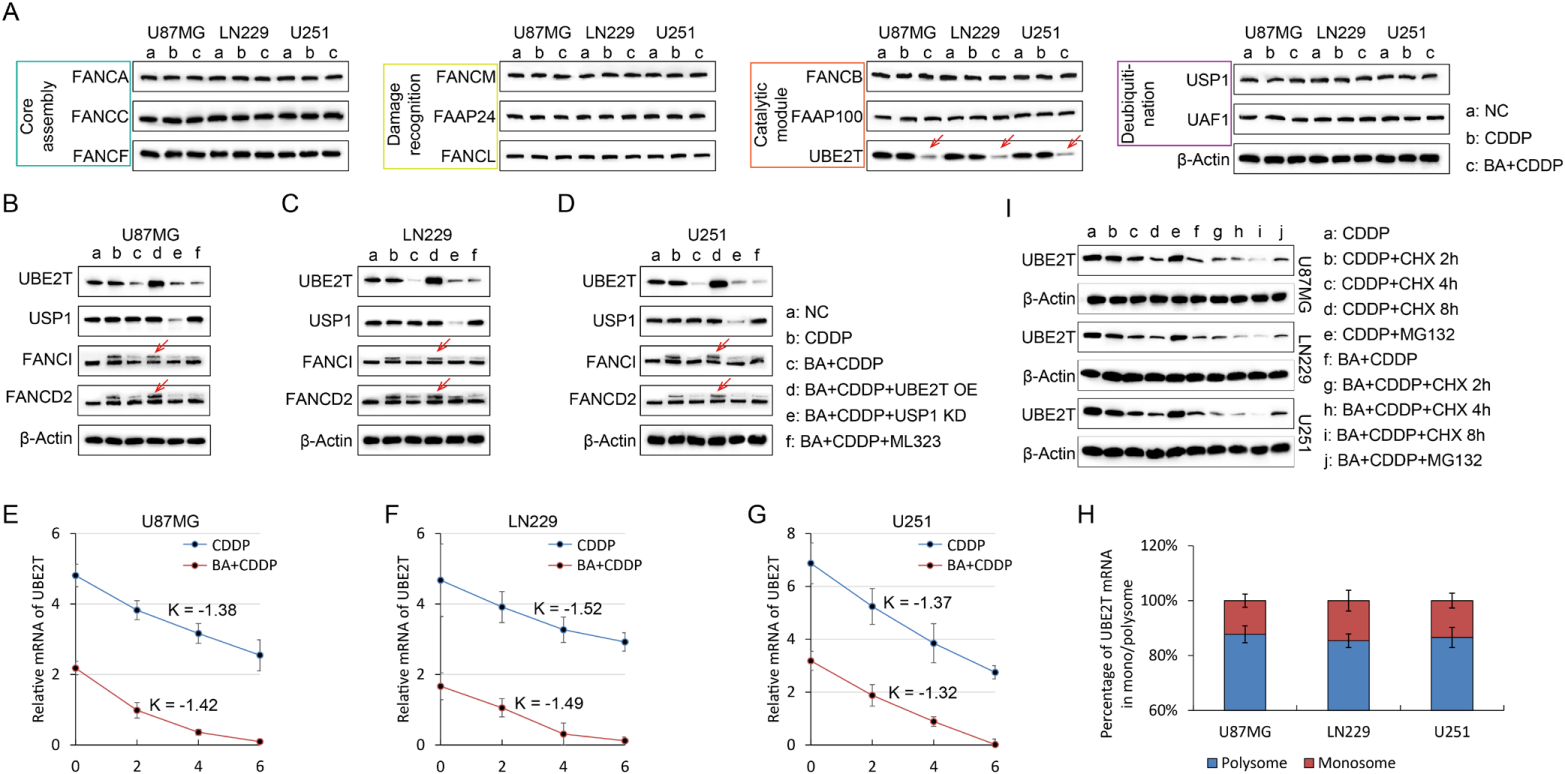
Ba selectively downregulates UBE2T expression through transcriptional suppression rather than mRNA stability, translation, or protein degradation. (A) WB assay show the expression of Fanconi anaemia (FA) pathway components in U87MG, LN229, and U251 glioma cells. Only UBE2T shows a marked reduction in protein abundance following BA pretreatment prior to cisplatin (CDDP) exposure (red arrows). (B–D) WB assay show that overexpression (OE) of UBE2T restores ID2 monoubiquitination suppressed by BA (red arrows), whereas USP1 knockdown (KD) or pharmacological inhibition with ML323 fail to rescue UBE2T levels in U87MG (B), LN229 (C), and U251 (D) glioma cells. β‐Actin served as a loading control. (E–G) Actinomycin D (ActD) chase assays show comparable mRNA decay kinetics (K values) for UBE2T in CDDP and BA + CDDP groups across U87MG (E), LN229 (F), and U251 (G) glioma cells, indicating unaffected mRNA stability. (H) Polysome profiling reveals similar proportions of UBE2T transcripts in polysome and monosome fractions, suggesting no translational defect. (I) Cycloheximide (CHX) chase and MG132 proteasome inhibition assays show no significant changes in UBE2T protein stability in BA‐treated cells, excluding post‐translational degradation as the cause of UBE2T reduction.

To pinpoint the stage at which BA interferes with ID2 complex monoubiquitination, we systematically evaluated proteins representing distinct functional modules of the FA signalling pathway, including FANCA, FANCC, and FANCF (core assembly), FANCM and FAAP24 (damage recognition), FANCL, FANCB, FAAP100 and UBE2T (catalytic module), as well as USP1 and UAF1 (deubiquitination). Among these, only UBE2T exhibited a pronounced reduction in protein abundance when glioma cells were pretreated with BA prior to cisplatin exposure (Figure 3A). Restoration of UBE2T expression by ectopic overexpression reinstated ID2 monoubiquitination even in the presence of BA, whereas USP1 depletion by siRNA or pharmacological inhibition with ML323 did not counteract the BA‐mediated suppression (Figure 3B–D). At the transcript level, qPCR analyses revealed that UBE2T mRNA was markedly downregulated by BA. However, Actinomycin D chase experiments indicated no difference in decay kinetics of UBE2T mRNA between BA‐treated and control groups (Figure 3E–G), suggesting that the reduction occurred at transcription rather than mRNA stability. Polysome profiling further demonstrated that UBE2T transcripts were equally distributed between monosome and polysome fractions regardless of BA exposure (Figure 3H), excluding impaired translation initiation or elongation. Additionally, neither cycloheximide (CHX) nor the proteasome inhibitor MG132 altered UBE2T protein levels in BA‐treated glioma cells (Figure 3I), thereby ruling out protein degradation. Together, these findings strongly support that BA primarily attenuates UBE2T expression at the transcriptional level.

**FIGURE 4:**
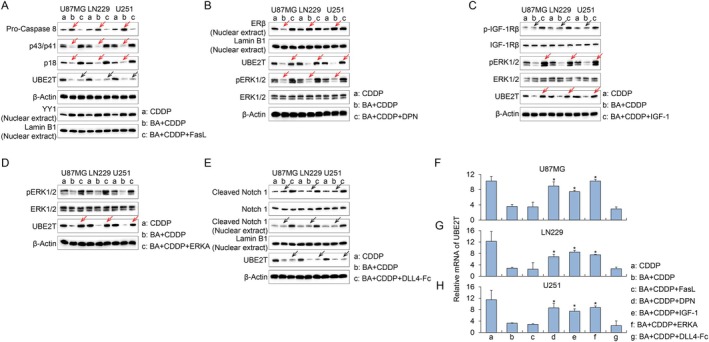
Effects of BA on YY1/FAS, ERβ, insulin/IGF‐1, MAPK/ERK, and Notch signalling pathways regulating UBE2T expression in glioma cells. (A) WB assay show that BA suppresses the YY1/FAS axis, decreasing cleaved caspase‐8 and YY1 nuclear accumulation (red arrows); FASL co‐treatment failed to restore UBE2T (black arrows). (B) WB assay show that BA inhibits ERβ nuclear localization and ERK1/2 phosphorylation (red arrows); ERβ activation by DPN re‐stimulated p‐ERK1/2 and elevated UBE2T (red arrows). (C) WB assay show that the insulin/IGF‐1 pathway is unaffected by BA (black arrows), but IGF‐1 treatment enhances IGF‐1Rβ phosphorylation, increases p‐ERK1/2, and up‐regulates UBE2T (red arrows). (D) WB assay show that direct ERK activation (ERK agonist) increases UBE2T expression (red arrows), confirming ERK as a convergent effector. (E) BA does not alter Notch signalling, and DLL4‐Fc–induced Notch activation shows no impact on UBE2T. β‐Actin served as loading controls of total protein, and Lamin B1 for nuclear extracts. (F–H) The relative mRNA levels of UBE2T are detected by qPCR in U87MG (F), LN229 (G) and U251 glioma cell lines (H) additionally treated with agonists of the pathway mentioned above. “*” indicates significant difference compared with BA+CDDP group.

Given that BA has previously been implicated in blocking diverse canonical YY1/FAS [27], ERβ [28], insulin/IGF‐1 [29], MAPK/ERK [30] and Notch [31] signalling pathways in different pathological models, we next investigated which pathway was responsible for UBE2T downregulation in glioma. To this end, cells were co‐treated with BA and specific agonists of these pathways to assess their capacity to rescue UBE2T expression. Western blot analysis demonstrated that BA differentially affected multiple signalling pathways regulating UBE2T expression in glioma cells (Figure 4A–E). In the YY1/FAS axis, BA treatment markedly decreased the levels of cleaved caspase‐8 (p43/p41, p18) and nuclear YY1 compared with cisplatin alone, whereas forced activation of the pathway by FASL failed to restore UBE2T expression (Figure 4A), suggesting that the YY1/FAS pathway can be suppressed by BA but is not the principal driver of UBE2T induction. In contrast, BA inhibited ERβ signalling, as evidenced by reduced nuclear ERβ accumulation and attenuated ERK1/2 phosphorylation. Pharmacological activation of ERβ with the agonist DPN re‐established p‐ERK1/2 and consequently increased UBE2T expression (Figure 4B). The insulin/IGF‐1 pathway was unaffected by BA: total and phosphorylated IGF‐1Rβ levels remained similar to cisplatin control, yet stimulation with IGF‐1 robustly enhanced p‐ERK1/2 and UBE2T expression (Figure 4C), indicating that insulin/IGF‐1 signalling can up‐regulate UBE2T via the MAPK/ERK cascade. Direct activation of ERK1/2 using an ERK agonist also elevated UBE2T expression (Figure 4D), confirming that ERK activity acts downstream of these upstream signals. Finally, BA had no detectable influence on Notch signalling, and DLL4‐Fc‐mediated Notch activation did not change UBE2T expression (Figure 4E). Consistently, qPCR assay also validated that activating MAPK/ERK pathway could rescue the UBE2T transcription suppressed by BA (Figure 4F–H).

### 3.3 BA Enhances the Antitumour Activity of Cisplatin in Glioma Xenografts

**FIGURE 5:**
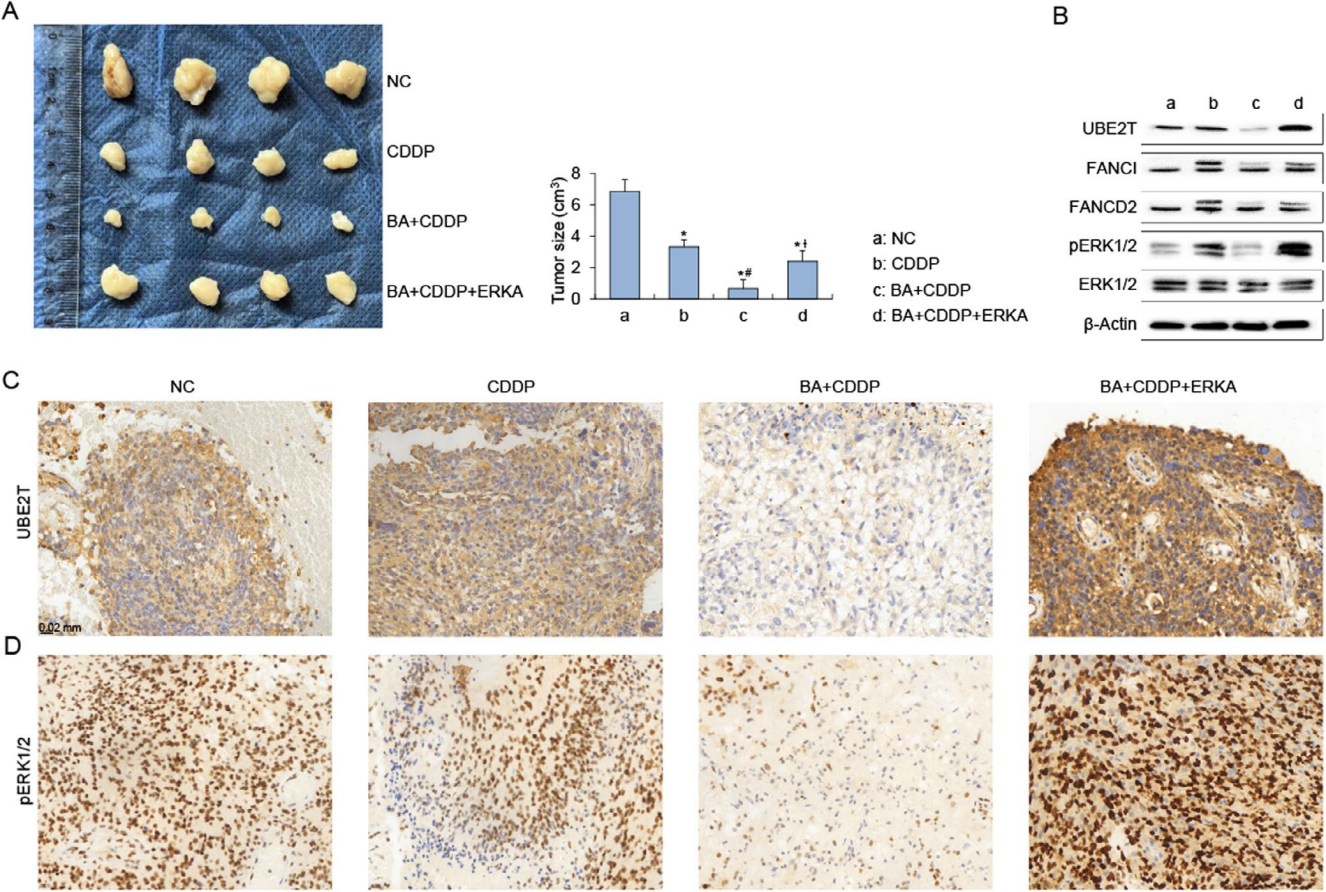
ERK activation reverses the tumour‐suppressive effect of BA in cisplatin‐treated xenografts. (A) Representative images of xenograft tumours and quantitative analysis of tumour volumes in four groups: Control (NC), cisplatin (CDDP), BA + CDDP, and BA + CDDP + ERK agonist (ERKA). BA + CDDP treatment markedly reduces tumour growth compared with CDDP alone, while ERK activation restores tumour size to near‐control levels. Data are shown as mean ± SEM; *p < 0.05 vs. NC, #*p* < 0.05 vs. CDDP, †*p* < 0.05 vs. BA + CDDP. (B) Western blot analysis of xenograft lysates showing decreased UBE2T as well as monoubiquitination of FANCI and FANCD2 in the BA + CDDP group, along with suppressed ERK1/2 phosphorylation; ERK agonist treatment restores p‐ERK1/2 and UBE2T expression. β‐Actin served as the loading control for total protein. (C, D) Immunohistochemical staining of xenograft tumour sections confirming reduced UBE2T (C) and p‐ERK1/2 (D) expression in the BA + CDDP group, with re‐elevation following ERK agonist co‐treatment. Scale bar = 20 μm.

Because the FA pathway is central to the repair of cisplatin‐induced DNA lesions, its inhibition was hypothesised to sensitise glioma cells to chemotherapy. To test this, a xenograft model was generated by subcutaneous implantation of U87MG cells in nude mice. Animals receiving combined treatment with cisplatin and BA displayed a pronounced reduction in tumour growth compared with those treated with cisplatin alone. Importantly, co‐administration of an MAPK/ERK agonist abolished the BA‐mediated tumour suppression, restoring tumour volumes to levels comparable with the control groups (Figure 5A).

At the molecular level, BA treatment led to impaired monoubiquitination of FANCI and FANCD2, accompanied by a significant decrease in UBE2T expression (Figure 5B). Consistently, MAPK/ERK signalling activity was suppressed, as indicated by reduced phosphorylation of ERK1/2 (Figure 5B). Immunohistochemical staining of tumour tissues corroborated these findings, showing diminished UBE2T (Figure 5C) and p‐ERK1/2 (Figure 5D) expression in xenografts from BA‐treated mice.

**FIGURE 6:**
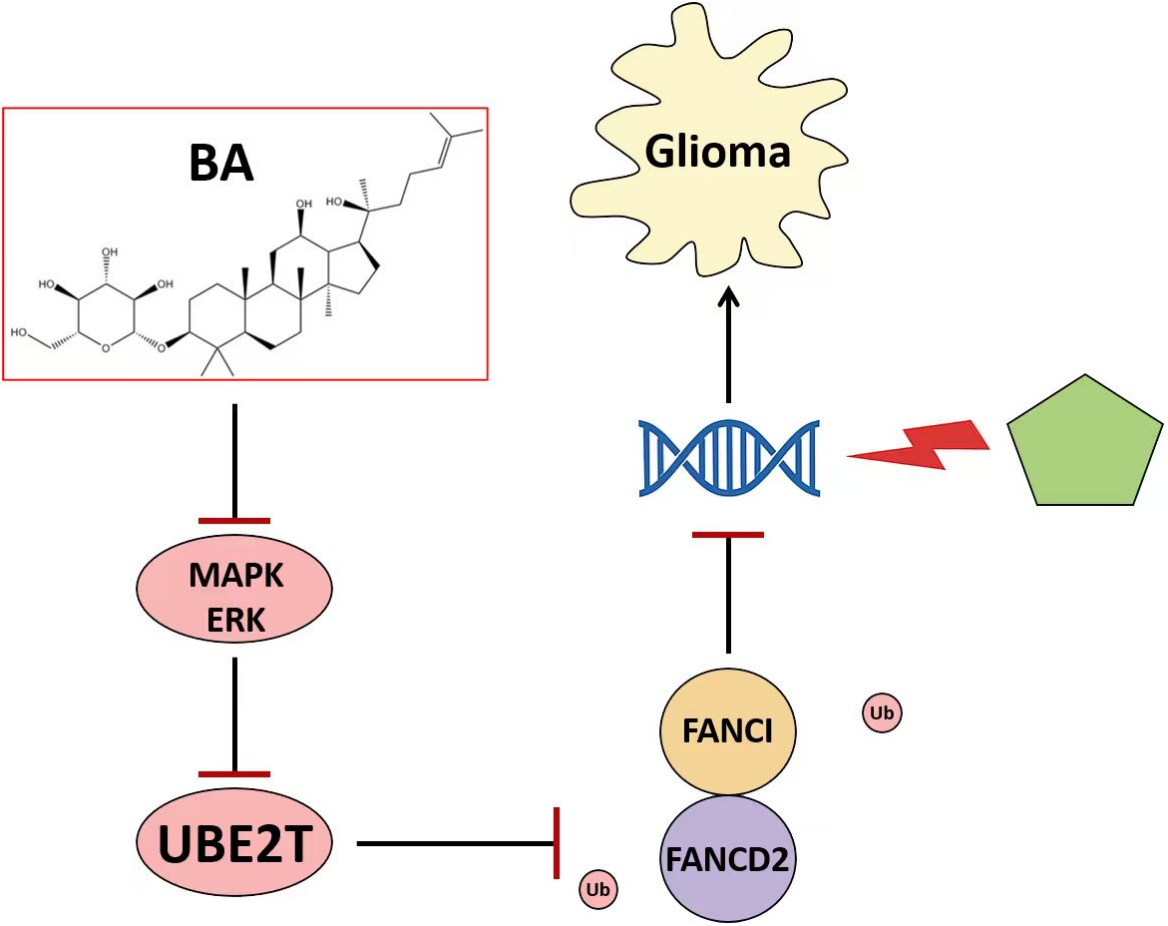
Schematic model illustrating how BA modulates MAPK/ERK signalling pathway to suppress UBE2T‐mediated DNA repair and glioma tumour growth responding to cisplatin.

Taken together, these results demonstrated that BA inhibited MAPK/ERK signalling, thereby lowering UBE2T levels, preventing FA pathway activation, and ultimately enhancing the cytotoxic response of glioma tumours to cisplatin in vivo (Figure 6).

## 4 Discussion

The Fanconi anaemia (FA) pathway constitutes a central genome maintenance system that orchestrates the repair of DNA interstrand crosslinks (ICLs) through the coordinated monoubiquitination of FANCI and FANCD2, followed by recruitment of repair effectors such as BRCA1, REV1, and ERCC1 [32]. Overactivation of this pathway contributes to platinum resistance in multiple malignancies, including glioma, ovarian, and bladder cancers [33]. Inhibition of FA signalling components—such as FANCL, UBE2T or FANCD2—has been shown to sensitise tumour cells to crosslinking agents without inducing catastrophic genomic instability. Our findings that BA attenuates cisplatin‐induced FANCI/FANCD2 monoubiquitination support the concept that pharmacologic modulation of FA transcriptional control, rather than enzymatic blockade, could be an effective and selective strategy to overcome chemoresistance. BA is a pentacyclic triterpenoid best known for its pro‐apoptotic and anti‐inflammatory properties, often through inhibition of NF‐κB [34] or PI3K/AKT signalling [35]. However, recent evidence indicates that BA and related compounds also influence nuclear transcriptional networks, including E2F1‐ and YY1‐regulated genes [27]. Our observation that BA suppresses UBE2T transcription adds a new mechanistic dimension to its pharmacology. Structurally similar triterpenoids such as oleanolic acid and ursolic acid have been shown to repress E2/E3 ligase expression and modulate histone acetylation [36, 37]. Therefore, BA may exert genome‐stabilising effects through epigenetic remodelling or transcriptional interference rather than direct DNA damage. Future promoter‐mapping studies, including chromatin immunoprecipitation (ChIP) for YY1 or E2F1 binding to the UBE2T promoter, would clarify whether BA's repression involves specific transcriptional repressors.

MAPK/ERK signalling plays a well‐established role in DNA repair gene transcription. ERK activation facilitates the expression of XRCC1, BRCA1 and FANCD2 through AP‐1 and ELK1 transcription factors [38, 39, 40]. Conversely, pharmacologic ERK inhibition attenuates DNA repair and sensitises tumours to genotoxic therapy [41, 42]. Our data showing that BA reduces ERK phosphorylation while suppressing UBE2T expression suggest that UBE2T is a downstream target of ERK‐dependent transcriptional control. In silico analyses from TCGA cohorts have revealed a positive correlation between phosphorylated ERK and UBE2T transcript levels in colorectal cancer [43] and lung adenocarcinoma [44]. Thus, we propose a model in which ERK activation sustains FA pathway readiness through transcriptional upregulation of UBE2T. Investigating whether ERK‐driven transcription factors such as ELK1 or c‐Fos directly occupy the UBE2T promoter will be crucial to confirm this regulatory hierarchy. Although our results indicate that MAPK/ERK activation enhances UBE2T expression, the precise nature of this regulatory relationship remains to be fully elucidated. Prior studies have reported the opposite directionality that UBE2T can stimulate ERK signalling in several tumour models [45], suggesting that the ERK‐UBE2T relationship may be context‐dependent or potentially bidirectional. Our current data support a model in which ERK functions upstream of UBE2T transcription in this cellular context, yet they do not exclude the possibility that UBE2T might also modulate ERK activity under different physiological conditions. Whether ERK directly targets transcriptional regulators of UBE2T or whether ERK activation induces a secondary signalling cascade that ultimately converges on UBE2T expression remains unknown. Future studies involving promoter mapping, chromatin accessibility profiling, and perturbation of specific ERK‐responsive transcription factors will be necessary to fully characterise this regulatory network.

Dual inhibition of ERK and FA signalling represents a rational therapeutic approach to reverse platinum resistance. MEK inhibitors such as trametinib and selumetinib have been shown to enhance cisplatin efficacy by repressing DNA repair gene transcription [46, 47]. BA appears to mimic this pharmacologic effect via a natural compound scaffold, potentially with improved tolerability. Importantly, the selective interference with interstrand, but not intrastrand, crosslink repair observed in our study may preserve essential base excision and nucleotide excision repair functions in normal cells. This selectivity could reduce systemic toxicity, an advantage over global DNA repair inhibitors like ATR or PARP inhibitors. Thus, BA's transcriptional suppression of UBE2T through ERK inhibition may represent a balanced way to exploit FA dependency in cancer cells.

Gliomas are notorious for intrinsic resistance to DNA‐damaging agents, largely due to constitutive activation of repair and survival pathways. High UBE2T expression has been correlated with poor survival and temozolomide resistance in glioblastoma [48, 49], while MAPK/ERK hyperactivation promotes replication stress tolerance and radioresistance [50]. Our findings suggest that BA disrupts this adaptive circuitry by co‐targeting both ERK and FA nodes. Similar network‐level interactions have been observed in other tumours: RAS/ERK signalling promotes HR gene expression in melanoma [51], and hypoxia‐induced ERK activation enhances FA pathway proteins in ovarian cancer [52]. These parallels reinforce a broader principle—oncogenic MAPK signalling operates as a transcriptional rheostat aligning metabolic stress with DNA repair capacity. Interfering with this coupling, as BA appears to do, could sensitise gliomas to crosslinking and radiomimetic drugs.

Although our data firmly link BA‐mediated UBE2T repression to MAPK/ERK inhibition, BA is a pleiotropic agent that likely affects multiple signalling axes. Potential off‐target effects on Notch, Wnt/β‐catenin, or NRF2 pathways cannot be excluded without comprehensive proteomic and phosphoproteomic analyses. Moreover, the observed chemosensitisation requires validation in additional glioma subtypes and in primary patient‐derived models. The safety margin for combining BA with cisplatin also warrants pharmacokinetic and toxicity assessment in normal astrocytes and glial progenitor cells. Finally, identifying the specific transcription factors or epigenetic modifiers bridging ERK activity and UBE2T transcription will further refine this therapeutic concept and may uncover novel druggable regulators of DNA repair homeostasis.

In conclusion, our study delineates a mechanistic axis wherein MAPK/ERK signalling transcriptionally sustains UBE2T expression and FA pathway activation, conferring platinum resistance in glioma. BA interrupts this axis by repressing ERK phosphorylation and UBE2T transcription, thereby impairing interstrand crosslink repair and enhancing cisplatin efficacy. This work highlights an unappreciated link between oncogenic signalling and genome maintenance, suggesting that transcriptional coupling between MAPK and FA pathways may be a key adaptive vulnerability in therapy‐resistant glioma.

These findings position BA as both a mechanistic probe and a potential lead compound for rational combination regimens that exploit ERK‐UBE2T‐FA dependency in aggressive tumours.

## Author Contributions

Y.B. performed all experiments and analysed all data. M.W. prepared all figures, drafted and revised manuscript.

## Funding

The authors have nothing to report.

## Conflicts of Interest

The authors declare no conflicts of interest.

## Data Availability

The data that support the findings of this study are available from the corresponding author upon reasonable request.
